# Silver nanoparticles: synthesis, characterisation and biomedical applications

**DOI:** 10.1515/biol-2020-0094

**Published:** 2020-11-19

**Authors:** Ahmad Almatroudi

**Affiliations:** Department of Medical Laboratories, College of Applied Medical Sciences, Qassim University, Buraydah, 51452, Saudi Arabia

**Keywords:** silver nanoparticles, biological synthesis, characterisation, antimicrobial agent, antibiofilm, health management activity

## Abstract

Nanotechnology is a rapidly growing field due to its unique functionality and a wide range of applications. Nanomedicine explores the possibilities of applying the knowledge and tools of nanotechnology for the prevention, treatment, diagnosis and control of disease. In this regard, silver nanoparticles with diameters ranging from 1 to 100 nm are considered most important due to their unique properties, ability to form diverse nanostructures, their extraordinary range of bactericidal and anticancer properties, wound healing and other therapeutic abilities and their cost-effectiveness in production. The current paper reviews various types of physical, chemical and biological methods used in the production of silver nanoparticles. It also describes approaches employing silver nanoparticles as antimicrobial and antibiofilm agents, as antitumour agents, in dentistry and dental implants, as promoters of bone healing, in cardiovascular implants and as promoters of wound healing. The paper also explores the mechanism of action, synthesis methods and morphological characterisation of silver nanoparticles to examine their role in medical treatments and disease management.

## 1 Introduction

Considering the epitome of scientific discoveries and inventions ever since the advent of man on Earth, the emergence of nanotechnology is a relatively recent development. However, the last 30 years have been witness to the invention of nanotechnology in the 1980s and its rise to prominence during the early 2000s with wide commercial applications in various sectors. Materials with unique properties are downsized to the level of individual atoms and molecules, collectively called nanoparticles, generally ranging from 1 to 100 nm. Potential uses for these particles include commercial, industrial, agricultural and medicinal applications. Although nanoparticles have an identical chemical composition to the parent material, physical properties including colour, strength, magnetic and thermodynamic properties and other physical aspects may differ widely. The application of nanomaterials in medicine is also a recent venture with most applications still in the research and development stage.

However, certain materials, due to their exemplary medicinal properties, have been part of the medicinal domain since time immemorial. Silver (Ag), due to its extraordinary range of bactericidal properties and therapeutic abilities, has been a part of medical treatment and management of various diseases since ancient times. It is a well-recognised fact that silver ions and silver-based compounds have a great microbial killing capacity [1,2]. However, the development of technologies and a better understanding of the mechanism of silver in disease prevention via killing of microorganisms have opened the door towards their uses in nanomedicine. Many approaches and methods have evolved for the effective synthesis of silver nanoparticles, including physical, chemical and biological techniques. While physical and chemical methods are commercially more cost-effective, the biological methods are relatively less harsh on the environment [3].

In nanomedicine, silver nanoparticles are extremely important due to their attractive physicochemical properties and biological functionality, including their high antimicrobial efficiency and relatively non-toxic, wide spectrum of bactericidal properties [4], anticancer properties and other therapeutic abilities, their unique ability to form diverse nanostructures [5] and their relatively low manufacturing cost [6].

Silver nanoparticles are intensively explored nanostructures ranging between 1 and 100 nm, primarily used for unconventional and enhanced biomedical applications in such areas as drug delivery, wound dressings, tissue scaffolding and protective coating applications. Moreover, the impressive available surface of nanosilver allows the coordination of many ligands, thus enabling tremendous possibilities with respect to the surface functionalisation of silver nanoparticles. Silver is routinely used in the form of silver nitrate (NO_3_^−^) for antimicrobial activity. In addition, silver nanoparticles are more beneficial as compared to free silver because their greater surface area increases the exposure of microbes. Furthermore, silver nanoparticles have emerged as a great field of interest for researchers because of their unique activity against a large range of microorganisms and due to resistance against commonly used antibiotics [7]. To date, several studies have reported applications in fields such as food processing, agriculture and agro-based industries, biomedical and medical remediation, healthcare products, consumer products, numerous industries, pharmaceuticals, in diagnostics, orthopaedics, drug delivery, imaging, filters as antitumour agents and as enhancer of tumour-killing effects of anticancer drugs.

The current review summarises the important approaches for the synthesis of silver nanoparticles as well as their various roles as antimicrobial and antibiofilm agents, antitumour agents, in dentistry, bone healing, dental implants, cardiovascular implants and wound healing.

## 2 Synthesis/production of silver nanoparticles

Several procedures are employed for the manufacture of silver nanoparticles, including physical, chemical and biological syntheses. It is worth noting that each method has its own advantages and disadvantages. During biological synthesis of silver nanoparticles, the organism acts as a capping agent, reducing agent or stabilising agent and reduces Ag^+^ to produce Ag^0^ [8]. Due to their low cost, high yields and low toxicity on the human body and the environment, biological methods based on natural products obtained from microorganism and plant sources have increased in popularity in recent years [9]. Different methods for synthesis of silver nanoparticles are described in the following sections.

### 2.1 Chemical methods

Various methods are available to synthesise silver nanoparticles. Chemical methods are beneficial because the equipment required is more convenient and simple than that used in biological methods. It has already been reported that silver ions receive electrons from the reducing agent and become converted into the metallic form, which finally aggregates to form silver nanoparticles. Among the silver salts used in chemical synthesis of silver nanoparticles, AgNO_3_ is one of the most commonly used due to properties such as low cost (Table 1) [10,11]. In 2002, Sun and Xia reported the synthesis of monodispersed silver nanocubes through reducing nitrate [12]. Mukherji and Agnihotri synthesised silver nanoparticles using AgNO_3_ as a precursor, and sodium borohydride and trisodium citrate as stabilising agents. It has been reported that sodium borohydride is a good reducing agent for the synthesis of silver nanoparticles having a size range of 5–20 nm. In comparison, trisodium citrate is the most effective reducing agent for the synthesis of silver nanoparticles of the size range 60–100 nm [13]. Polyvinylpyrrolidone (PVP) as a size controller and a capping agent, with ethylene glycol as a solvent and a reducing agent, is reported to give rise to silver nanoparticles with an average size less than 10 nm [14]. Patil et al. confirmed the synthesis of silver nanoparticles using hydrazine hydrate as the reducing agent and polyvinyl alcohol as the stabilising agent. Their results revealed that the resultant nanoparticles had a spherical morphology and these particles showed significant applications in biotechnology and biomedical science [15]. According to another important study, the synthesised silver nanoparticles were found to be spherical with different sizes [16].

The AgNO_3_ solution is heated to the reaction temperature in the precursor heating method and the nanoparticle size is observed to be most affected by the ramping rate, whereas in the precursor injection method, a silver nitrate aqueous solution is injected, and the reaction temperature is a key factor for the reduction of particle size and for achieving monodispersity [17]. High yield is the main advantage of chemical methods, compared to physical methods. Chemical methods are highly expensive, and chemicals and compounds used for silver nanoparticle synthesis such as borohydride, 2-mercaptoethanol, citrate and thio-glycerol are hazardous and toxic. It is extremely difficult to produce silver nanoparticles with a definite size and it requires an additional step to prevent particle aggregation [18]. Numerous hazardous and toxic by-products are produced during synthesis. Moreover, the reducing agents used in these methods are toxic [19].

### 2.2 Physical methods

Physical methods for the preparation of silver nanoparticles include evaporation–condensation and laser ablation. The main drawbacks of these methods are the huge amount of energy required, plus long duration for completion of the whole process.

Lee and Kang have reported that thermal decomposition of Ag^+^–oleate complexes results in the synthesis of monodispersed silver nanocrystallites [20]. In a study conducted by Jung et al., a small ceramic heater was used to prepare metal nanoparticles through evaporation/condensation processes. It was noticed that a constant temperature of the heater surface with time generated polydispersed nanoparticles. These silver nanoparticles were spherical and non-agglomerated [21]. Recently, it has been demonstrated that the polyol process produces spherical nanoparticles with different sizes under laser ablation [17,22]. To examine the effects of laser wavelength on the particle size, silver nanoparticles were synthesised through ablation with different lasers and it was noticed that decrease in laser wavelength reduced the average diameter of particles from 29 to 12 nm [23]. Nanosized particles of silver were prepared by Tsuji et al. through laser ablation in water to compare the formation efficacy and the size of colloidal particles produced by femtosecond pulses with colloidal particles produced by nanosecond laser pulses. The formation efficiency for femtosecond pulses was significantly lower than that for nanosecond pulses. Besides this, the size of colloids prepared via femtosecond pulses was less dispersed than that of colloids prepared by nanosecond laser pulses [24]. Seigal and colleagues examined the synthesis of silver nanoparticles through a direct physical deposition of metal into the glycerol. This approach was found to be a good alternative for time-consuming chemical processes. Furthermore, consequential nanoparticles were resistant to aggregation and had a narrow size distribution [25]. Speed, no requirement for toxic reagents and radiation utilised as a reducing agent are the advantages of physical methods of production. Solvent contamination, minimal yield, non-uniform distribution and high energy consumption are the disadvantages of physical methods (Table 2) [26].

**Table 1:** Chemical methods for the synthesis of monodispersed and quasi-spherical silver nanoparticles [11]

Reducing agent	Precursor agent	Capping agent	Experimental conditions
Trisodium citrate	Silver nitrate	Trisodium citrate	Diameter ≈ 10–80 nm; temperature ≈ boiling point
Ascorbic acid	Silver nitrate	Daxad 19	Diameter ≈ 15–26 nm; temperature ≈ boiling point
Alanine/NaOH	Silver nitrate	DBSA (dodecylbenzenesulfonic acid)	Diameter ≈ 8.9 nm; temperature ≈ 90°C; time ≈ 60 min
Ascorbic acid	Silver nitrate	Glycerol/PVP	Diameter ≈ 20–100 nm; temperature ≈ 90°C
Oleic acid	Silver nitrate	Sodium oleate	Diameter ≈ 5–100 nm; temperature ≈ 100–160°C; time ≈ 15–120 min
Trisodium citrate	Silver nitrate	Trisodium citrate	Diameter ≈ 30–96 nm; temperature ≈ boiling point; pH ≈ 5.7–11.1
Trisodium citrate	Silver nitrate	Trisodium citrate/Tannic acid	Diameter ≈ 10–100 nm; temperature ≈ 90°C

### 2.3 Biological methods

Production of silver nanoparticles by physical and chemical processes is expensive, time consuming and eco-unfriendly. Hence, it is very important to develop an environmentally and economically friendly method, which does not involve toxic chemicals [32] and avoids the other problems associated with chemical and physical means of production. Biological methods fill these gaps and have various applications in health management through regulation of various biological activities. Biological production methods include the use of fungi, bacteria and yeasts as well as plant sources. These sources make this approach very popular for medical applications of nanoparticles.

**Figure 1:**
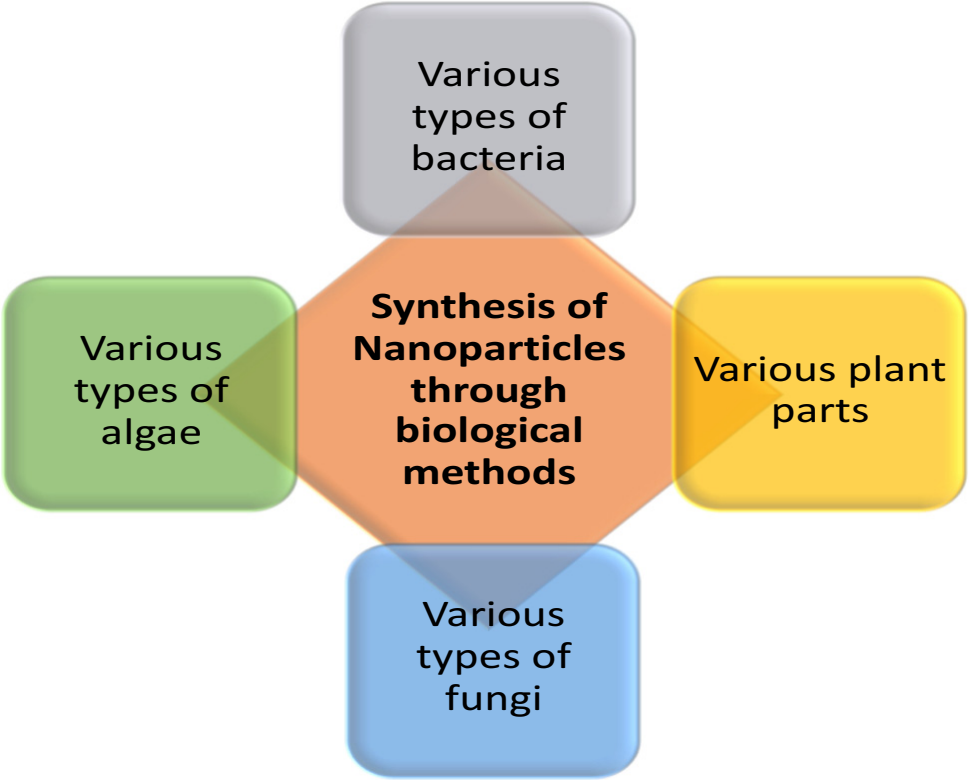
Different biological methods for the synthesis of silver nanoparticles.

It has been reported that nanoparticle production methods based on microorganisms and plants are safe, economic and are relatively less harmful to the environment than chemical synthesis [33,34]. Moreover, microorganisms and plants are able to absorb and accumulate inorganic metallic ions from their surrounding environment [35]. Biological production of silver nanoparticles mainly involves the use of microorganisms and plant sources (Figure 1) [36].

**Table 2:** Physical and chemical syntheses of silver nanoparticles

Type	Reducing agent	Biological activity	Characterisation	Ref.
Polydiallyldimethylammonium chloride and polymethacrylic acid capped silver nanoparticles	Methacrylic acid polymers	Antimicrobial	UV-Vis, reflectance spectrophotometry	[27]
Silver nanoparticles	Ascorbic acid	Antibacterial	UV-Vis, EFTEM	[28]
Chitosan-loaded silver nanoparticles	Polysaccharide chitosan	Antibacterial	TEM, FTIR, XRD, DSC, TGA	[29]
Silver nanoparticles	Hydrazine, d-glucose	Antibacterial	UV-Vis, TEM	[30]
PVP-coated silver nanoparticles	Sodium borohydride	—	UV-Vis, TEM, EDS, DLS, FIFFF	[31]

Abbreviations: UV-Vis – ultraviolet-visible spectroscopy, FIFFF – flow field-flow fractionation, DSC – differential scanning calorimetry, TEM – transmission electron microscopy, EDS – energy-dispersive spectroscopy, EFTEM – energy filtered TEM, FTI R – Fourier transform infrared, DLS – dynamic light scattering, XRD – X-ray diffraction, TGA – thermogravimetric analysis.

#### 2.3.1 Production in bacteria

Recently, a study was performed to produce silver nanoparticles through the reduction of aqueous Ag^+^ ions using the culture supernatants of various bacteria. This approach was demonstrated to be fast and the interaction of silver ions with the cell filtrate generated silver nanoparticles within 5 min. Moreover, this study also reported that piperitone partially inhibited the reduction of Ag^+^ to metallic silver nanoparticles [37]. It is important to note that the nitro reduction activity of *Enterobacteriaceae* is inhibited by the natural product piperitone. It is assumed that the bioreduction of silver ions to silver nanoparticles might be partially inhibited by different strains of *Enterobacteriaceae* such as *Klebsiella pneumoniae*. Korbekandi and colleagues studied the optimisation of silver nanoparticle production by *Lactobacillus casei* subspecies *casei*, confirming the bioreductive synthesis of silver nanoparticles [38]. Liu et al. showed the formation of nanoparticles from dried cells of *Bacillus megaterium* [39]. Das et al. have described the extracellular synthesis of silver nanoparticles through a bacterial strain. The study showed that the treatment of *Bacillus* strain CS 11 with AgNO_3_ resulted in the formation of silver nanoparticles extracellularly [40].

#### 2.3.2 Synthesis/production based on fungi

Various types of fungi have been reported to be involved in the production of silver nanoparticles [41]. The production of silver nanoparticles by fungi has been found to be very quick. Many researchers have studied the biosynthesis of silver nanoparticles by fungi in detail [32]. One study has shown the extracellular biosynthesis of spherical silver nanoparticles by interaction of *Fusarium solani* with silver nitrate [42]. Syed and colleagues have reported the biosynthesis of silver nanoparticles by the *Humicola* sp. It was shown that a precursor solution was reduced by the interaction between *Humicola* sp. and Ag^+^ ions and extracellular nanoparticles were produced [43]. Owaid and colleagues have reported the production of silver nanoparticles by the bioreduction of silver nitrate induced by the extract of *Pleurotus cornucopiae* [44]. Xue et al. conducted an experiment to biosynthesise silver nanoparticles with antifungal properties using *Arthroderma fulvum* [45]. Vigneshwaran et al. reported that the interaction of silver nitrate solution with the fungus *Aspergillus flavus* resulted in the accumulation of silver nanoparticles on the surface of its cell wall [46]. Furthermore, Bhainsa and D’Souza had investigated the extracellular biosynthesis of silver nanoparticles using *Aspergillus fumigatus*. The results indicated that the interaction of silver ions with the cell filtrate generated silver nanoparticles in a very short time [47]. However, using *Fusarium oxysporum* results in an extracellular production of silver nanoparticles with a size of 5–50 nm [48]. Additionally, incubation of *Phanerochaete chrysosporium* mycelium with silver nitrate solution produced silver nanoparticles [49]. Korbekandi and colleagues showed the bioreductive production of silver nanoparticles by using *Fusarium oxysporum* [50].

#### 2.3.3 Production in algae

This approach is a feasible substitute for physical and chemical methods of nanoparticle production because it is economic and eco-friendly [51]. Furthermore, algae have a high capacity for metal uptake. It has been seen that biological sources such as marine algae have the capacity to catalyse specific reactions. This capacity is key to modern and realistic biosynthetic plans [52]. A study based on the algae extract has shown that the change of colour from yellow to brown can indicate the reduction of silver ions to silver nanoparticles. In addition, Rajeshkumar and colleagues noticed the deep brown colour of silver nanoparticles at 32 h and it was observed that the time of incubation was directly associated with the increase in colour intensity [53]. Silver nanoparticles were synthesised through the reduction of aqueous solutions of silver nitrate with powder and solvent extracts of *Padina pavonia*. Additionally, the achieved nanoparticles showed high stability, fast formation and small size [54]. Salari and colleagues reported the production of silver nanoparticles through bioreduction of silver ions induced by *Spirogyra varians* [55].

#### 2.3.4 Production in yeast

Yeasts have been reported to have the capability to produce silver nanoparticles. In addition, silver nanoparticle production methods based on yeast are cost-effective as well as eco-friendly. In this regard, Niknejad and colleagues performed a study that was based on *Saccharomyces cerevisiae*. It was noted that with increasing time of incubation, the colourless sample slowly turned to reddish-brown after adding Ag^+^ ions to the yeast culture. Furthermore, the colour of the solution changed into strong reddish-brown [56]. In 2003, Kowshik et al. have reported the extracellular synthesis of nanoparticles through the interaction of soluble silver with a silver-tolerant yeast in its log phase of growth [57].

#### 2.3.5 Synthesis based on plants/plant extracts

Like other biological methods, production in plants is better than chemical and physical methods because high temperature, energy and toxic chemicals are not needed and it is cost-effective and environment-friendly [58]. Numerous active constituents are present in *Aloe vera* leaves. These ingredients include lignin, hemicellulose and pectins, which have been shown to have a clear role in the reduction of silver ions [59]. In a recent study, silver nanoparticles were synthesised using an aqueous solution of the plant extract of Saudi Arabia *Origanum vulgare* L. The result demonstrated that synthesis of silver nanoparticles occurred by reduction of Ag^+^ ions. During this process, the colour of the reaction mixture was converted from light brown to dark brown. On the other hand, in the absence of plant extract no change in colour was observed under the same conditions [60]. The results of another study reported that the colour of the aqueous silver nitrate solution was changed from faint light to yellowish brown after the addition of different concentrations of aqueous leaf extracts of *Azadirachta indica* [61]. López-Miranda et al. biosynthesised silver nanoparticles rapidly by using *Tamarix gallica* plant extract [62].

Chinnappan et al. have reported a fast and simple method for the synthesis of silver nanoparticles using an extract of *Bauhinia purpurea* flower [63]. In 2016, Ibraheim et al. reported the synthesis of silver nanoparticles from silver nitrate using aqueous pomegranate juice extract as a reducing agent and their results demonstrated that the use of juice extract leads to a quick synthesis of silver nanoparticles from AgNO_3_ solution. It was found that the colour changed from light yellow to reddish-brown with the formation of silver nanoparticles after exposure to microwaves for a few minutes [64]. Lakshmanan et al. synthesised silver nanoparticles using *Cleome viscosa* plant extract and the study revealed that extract of this plant has a good ability to reduce silver nitrate into metallic silver [65].

Prasad et al. employed aqueous leaf extracts of *Moringa oleifera* to develop a simple and quick method for bioreduction of silver nanoparticles. Their findings concluded that *Moringa oleifera* had a strong potential for synthesis of silver nanoparticles via rapid reduction of silver ions [66]. In this regard, another finding indicated a fast and convenient method for the synthesis of silver nanoparticles using *Ficus benghalensis* leaf extract and the reduction of silver ions into silver nanoparticles occurred within short periods (5 min) of reaction time without using any hard conditions [67]. Moreover, the treatment of aqueous solutions of silver nitrate and chloroauric acid with neem leaf extract leads to the fast synthesis of stable silver and gold nanoparticles at high concentrations [68]. Earlier investigators are accountable for pioneering nanoparticle synthesis through using plant extracts [60,68,69,70,71,72].

Ponarulselvam et al. concluded that extracts of the leaves of *Catharanthus roseus* could be used in the synthesis of silver nanoparticles that exhibited antiplasmodial activity against *Plasmodium falciparum* [73]. Some studies have reported that silver ions are reduced extracellularly in the presence of fungi to generate stable silver nanoparticles in water [42,74]. Zarghar and colleagues have indicated the formation of spherical silver nanoparticles by using methanolic leaf extracts of *Vitex negundo* and demonstrated the antibacterial activity of these silver nanoparticles against both Gram-positive and Gram-negative bacteria [75].

#### 2.3.6 Synthesis based on DNA

DNA can be used as a reducing agent for silver nanoparticle synthesis. High affinity of silver ions with DNA base pairs makes DNA a template stabiliser. Synthesised silver nanoparticles were found at N-7 phosphate and guanine base pair on DNA strand. Another study reported the synthesis of silver nanoparticles with calf thymus DNA [36,76].

## 3 Characterisation techniques for nanoparticles

**Figure 2:**
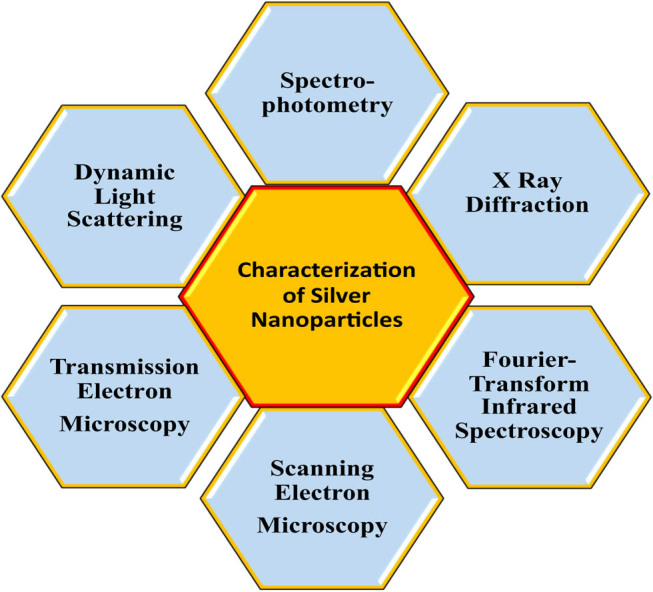
Various techniques used for characterisation of silver nanoparticles.

Characterisation is an important step in the green synthesis of nanoparticles. It is a pivotal step to determine the morphology, surface chemistry, surface area and disparity in the nature of any silver nanoparticle. Various techniques are used for characterisation of silver nanoparticles (Figure 2).

### 3.1 UV-Vis spectrophotometry

This technique is most widely utilised to characterise metallic nanoparticles by monitoring their stability and synthesis [77]. The synthesis of a metallic nanoparticle from its particular salt provides a characteristic peak with strong absorptions in the visible region [78]. Various studies have revealed that, in general, the absorption band at around 200–800 nm wavelength is best for the characterisation of particles in the size range of 2–100 nm [79]. The valence and conduction bands in silver nanoparticles are very close to each other. Electrons move freely in these bands and give rise to a surface plasmon resonance absorption band. The silver nanoparticle’s absorption depends upon the chemical surroundings, dielectric medium and particle size. Examination and study of the surface plasmon peak is well known for several metal nanoparticles having a size range of 2–100 nm. Stability of silver nanoparticles produced through biological methods was examined for about 12 months and a surface plasmon resonance peak at the same wavelength was found using UV-Vis spectrophotometry [18].

### 3.2 X-ray diffraction analysis (XRD)

XRD is an analytical technique broadly used to observe the structure of crystalline metallic nanoparticles by penetration of X-rays deeply into the material [80,81]. The resulting diffraction pattern confirms the formation of nanoparticles with crystalline structure [82].

To calculate the particle size from the XRD data, the Debye–Scherrer equation is applied by determining the width of the Bragg reflection law according to the equation: *d* = *Kλ*/*β* cos *θ*, where *d* is the particle size (nm), *K* is the Scherrer constant, *λ* is the wavelength of X-ray, *β* is the full width half maximum and *θ* is the diffraction angle (half of Bragg angle) that corresponds to the lattice plane [83].

Therefore, the structural features of various materials, such as biomolecules, polymers, glasses and superconductors, can be examined by XRD [18]. Moreover, XRD is a potent method for the study of nanomaterials [84].

### 3.3 Fourier transform infrared spectroscopy (FTIR)

FTIR can be utilised to explore the surface chemistry of synthesised metal nanoparticle and to observe the involvement of biomolecules in nanoparticle synthesis [80] and can be used for analysing different capping agents.

In FTIR, infrared rays are passed through the sample, some are absorbed by the sample and the remaining pass through it. The resulting spectra indicate the absorption and transmission that are characteristic of the sample material [85]. FTIR is a cost-effective, appropriate, simple and non-invasive technique to determine the function of biological molecules in the reduction of silver nitrate to silver [18].

### 3.4 Energy-dispersive X-ray spectroscopy (EDX)

EDX is an important technique for the analysis of the elemental composition of a sample and its application to nanotechnology has been documented. All elements have different atomic structures producing a unique set of peaks in the X-ray spectrum [86] and these can be used to study the elemental composition of any nanoparticle.

### 3.5 Scanning electron microscopy (SEM)

The topography and morphology of nanoparticles can be observed by SEM, which is also used to calculate the size of various nanoparticles at the micro- (10^−6^) and nano (10^−9^) scales [87,88]. A high-energy electron beam, produced by SEM, is directed at the surface of the sample nanoparticles and the backscattered electrons produced give the characteristic features of the sample [89]. Electron microscopy analysis is used to examine the changes in the morphology of the cell before and after nanoparticle treatment. Several studies have reported that the visible modifications in cell shape and perforations of nanoparticles in the cell wall have been used as indicators of the antimicrobial action of nanoparticles [90,91]. Using SEM, control bacterial cells exhibited smooth and undamaged structures, while cells treated with silver nanoparticles for 60 min were significantly damaged, with clear morphological changes to the cell membrane leading to loss of membrane integrity [92].

### 3.6 Transmission electron microscopy (TEM)

TEM is a very useful technique for characterisation of nanoparticles, which provides information on size and morphology of nanoparticles [80,93]. TEM has a 1,000-fold higher resolution compared with SEM [94] and its images give more exact information related to size, shape and crystallography of the nanoparticles [81].

### 3.7 Dynamic light scattering (DLS)

DLS is a well-recognised technique for measuring the size and size distribution of molecules. It has been used to measure the size of nanoparticles and it is commonly used to characterise nanoparticles. Moreover, DLS has been extensively employed for sizing magnetic nanoparticles in the liquid phase [95,96] and its role in characterisation of various types of nanoparticles has been documented. The size of nanoparticle determined by DLS is generally larger than TEM due to the effect of Brownian motion. This technique can be used to determine the average size of nanoparticles in liquids [18].

### 3.8 Auger electron spectroscopy (AES)

AES is a surface‐sensitive analytical technique that derives from the interaction of an electron beam and atoms in residence at the surface of a sample [97] and is an outstanding analytical method for nanotechnology [98]. The oxidation state of silver as a component of a hybrid substance can be probed by AES [99].

### 3.9 Low-energy ion scattering (LEIS)

LEIS is a commonly used surface analytical technique, which is well recognised for its supreme surface sensitivity. With the help of this technique, the structure and the elemental composition of a given sample can be deduced [100–102]. Moreover, high sensitivity LEIS is a valuable surface analytical method for the characterization of SAM-functionalised nanomaterials [103].

## 4 Factors influencing the synthesis of silver nanoparticles

Shape, size and morphology of nanoparticles depend on physical and chemical factors which affect the synthesis of silver nanoparticles. In general, the basic parameters affecting the formation of silver nanoparticles include the following:

- methods of production
- temperature
- pH
- time
- shape and size.

### 4.1 Methods for the production of silver nanoparticles

There are many methods to manufacture nanoparticles, including physical and chemical techniques and biological protocols. Various organic or inorganic chemicals as well as living organisms are used for the synthesis of nanoparticles in these methods [104]. It has already been discussed that green synthesis is preferable to other methods because it is eco-friendly and cost-effective. Furthermore, green synthesis does not use high temperature, energy and toxic chemicals [58].

### 4.2 Temperature

Temperature has been found to be an important factor for the production of nanoparticles. Spherical nanoparticles are synthesised in the presence of elevated temperature. In contrast, nanotriangle formation occurs mostly at lower temperatures [90]. It has been shown that increase in temperature between 30 and 90°C boosts the frequency of synthesis [89,105] and sometimes also encourages the formation of smaller silver nanoparticles [106]. There are multiple reports suggesting that 25–37°C (room temperature) is the optimal range for the biogenic synthesis of metal nanoparticles.

### 4.3 pH

Most studies suggest that nanoparticle stability is improved in basic media than acidic [107,108]. However, a very high pH (pH > 11) was found to have some drawbacks such as the formation of agglomerated and unstable silver nanoparticles [109]. Therefore, it can be concluded that the shape and size of nanoparticles are determined by the pH.

### 4.4 Time

Decreasing the reaction time (minutes–hours) is another factor affecting the reduction of ions to a bulk metal with variant shapes. The optimum time period results in higher concentrations of nanoparticles in the medium, indicated by high absorbance peaks. Rai and colleagues have suggested that shape, size and optical properties of anisotropic nanoparticles can be fine-tuned by varying temperature. It was determined by employment of varying growth conditions and formation of different sizes of nanoparticles such as spherical, triangular, hexagonal and rectangular [70].

### 4.5 Shape and size

The shape and size of nanoparticles are crucial in determining their properties. It has been concluded that optimal activities are determined by the shape and size of the nanoparticle and most properties of nanoparticles are size-dependent [110].

## 5 Applications of silver nanoparticles in various industries

Silver nanoparticles have many properties making them desirable materials for a variety of industries, such as antibacterial and optical properties, availability and low production, processing and storage costs [111]. Furthermore, silver nanoparticles with a diameter of about 100 nm are very important for large-scale industries due to their small particle size, high surface area, quantum confinement and spread without agglomeration [112]. For these reasons, silver nanoparticles are used as alternatives in the manufacture of widely used goods and industries. Nowadays, silver nanoparticles are being explored in various industries such as medicine, biotechnology, material science and energy sectors, and particularly medicinal goods (wound dressings, medication delivery, biosensors and medical diagnostics, orthopaedics), the food and textile industries and water disinfection systems [113]. Furthermore, silver nanoparticles are often used for commercial products such as cosmetics and food processing as an essential additive. Furthermore, nanoparticles have many important uses, including spectrally sensitive solar energy absorption coatings and intercalation content for electrical batteries, optical receptors, polarising filters, chemical reaction catalysts, biolabelling and as antimicrobial agents [114]. In the agricultural sector, the utilisation of nanoparticles contributes to addressing the food security challenges raised by climate change. In the field of medicine [115], silver nanoparticles have introduced a new dimension to wound dressing and artificial implants and to the prevention of post-operative microbial contamination [116]. Silver nanoparticles are highly relevant as antibacterial agents in the textile, health and food industries. As an antibacterial agent, silver nanoparticles have various applications such as in the treatment of water, home appliances and sterilising medical equipment. Furthermore, silver nanoparticles are used in several textile goods (Table 3) [112]. The utilisation of preservatives can also be decreased due to the utilisation of silver nanoparticles [117].

**Table 3:** Use of silver nanoparticles in different industries

Industry/applications	Uses of silver nanoparticles
Pharmacological uses	Larvicidal
	Antimicrobial
	Wound healing
Textile	UV-ray blocking
	Medicinal devices and textiles
Water treatment	Potable water
	Ground water disinfection
	Wastewater disinfection
Biomedical industry	Antiviral
	Antibacterial
	Anti-inflammatory
	Antifungal
	Anticancer
Food industry	Food processing
	Food packaging

### 5.1 Uses of silver nanoparticles in biomedical sciences

**Figure 3:**
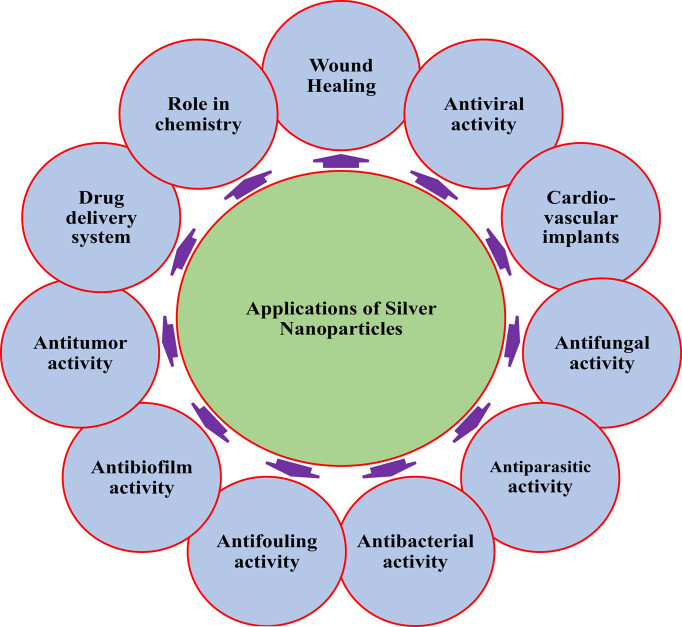
Applications of silver nanoparticles.

Silver nanoparticles play an important role in the modulation of various activities such as antimicrobial, antibiofilm, antiparasitic, antifouling, anticancer, antiviral and drug-delivery systems. Applications of silver nanoparticles are presented in Figure 3.

#### 5.1.1 Antiviral activity

Nanoparticles provide an alternative to drugs for treating and controlling the growth of viral pathogens. Biosynthesis of silver nanoparticles could result in potent antiviral agents to restrict virus functions. Suriyakalaa et al. studied bio-silver nanoparticles with convincing anti-HIV actions at an early stage of the reverse transcription mechanism [118]. Biosynthesised metallic nanoparticles have multiple binding sites for gp120 of the viral membrane to control the function of the virus. While another study reported that bio-based nanoparticles act as effective virucidal agents against free HIV or cell-associated virus [119]. Silver nanoparticles have been demonstrated to exert antiviral activity against HIV-1 at non-cytotoxic concentrations. These silver nanoparticles were evaluated to elucidate their mode of antiviral action against HIV-1 using a panel of different *in vitro* assays [120]. Another study reported the antiviral activity of silver nanoparticles with or without a polysaccharide coating against monkeypox virus. This study found that silver nanoparticles meaningfully inhibit monkeypox virus infection *in vitro* [121].

Exposing Tacaribe virus to silver nanoparticles prior to infection facilitated virus uptake into the host cells, whereas it was noticed that silver-treated virus showed significant reduction in viral RNA production and this finding demonstrated that silver nanoparticles are capable of inhibiting arenavirus infection *in vitro* [122]. Another study result showed that among the three types of silver nanoparticle-MHCs tested, Ag30-MHCs displayed the highest efficacy for viral inactivation [123].

#### 5.1.2 Antifungal activity

Silver nanoparticles have been shown to possess antifungal activity towards different fungi [124,125], but the mechanism behind it has not been fully understood. Silver nanoparticles have a tendency to disturb the structure of the cell membrane. This damaging effect on the membrane integrity and inhibition of the budding process has been suggested as the mechanism for antifungal activity of silver nanoparticles against *Candida albicans* species [126]. In a study of antibacterial and antifungal activity, nano-Ag sepiolite fibres containing monodispersed silver nanoparticles were used as the source of silver. Low melting soda lime glass powder containing nanoparticles had a good antibacterial and antifungal activity [127]. A study demonstrated that fluconazole in combination with silver nanoparticles showed the highest inhibition against *Candida albicans*. In this study, *Alternaria alternata* fungus was used for the extracellular biosynthesis of silver nanoparticles [128]. It was established that the concentration of silver nanoparticles between 30 and 200 mg/L significantly decreased the growth of fungi [129]. Furthermore, cell culture supernatant of strain GP-23 was used to synthesise silver nanoparticles and the biosynthesised silver nanoparticles showed a powerful antifungal activity [130]. *Trichoderma harzianum* cell filtrate was applied in the production of silver nanoparticles which resulted in their production within 3 h and TEM analysis demonstrated ellipsoid and spherical nanoparticles having a size range of 19–63 nm and an average size of 34.77 nm [131].

Jalal et al. concluded by the TEM analysis that the treatment of *Candida* cells with silver nanoparticles resulted in an extreme deformation of cells. Furthermore, the cell contraction was enhanced due to the interaction of nanoparticles with the fungal cell wall and membrane. It resulted in the disturbance of the structure of the cell membrane and inhibited the normal budding process due to the destruction and loss of membrane integrity [132]. Jalal et al. further showed the antimicrobial effects of *Syzygium cumini*-derived silver nanoparticles against *Candida* species and concluded that these nanoparticles have the capability to suppress the multiplication, germ tube and biofilm formation as well as secretion of hydrolytic enzymes by *Candida* species [133].

#### 5.1.3 Antiparasitic action

Silver nanoparticles have been found to possess larvicidal activities against the dengue vectors *Aedes aegypti* [134] and *Culex quinquefasciatus* [135]. Allahverdiyev et al. conducted a study to evaluate the effects of silver nanoparticles on biological parameters of *Leishmania tropica*. This study confirmed that silver nanoparticles possess antileishmanial effects due to their potential to inhibit the proliferation activity of promastigotes. Furthermore, silver nanoparticles were found to inhibit the survival of amastigotes in host cells, and this effect was increased in the presence of UV light [136]. Saad and colleagues synthesised silver and copper nanoparticles and tested their antiparasitic activity, finding that silver nanoparticles significantly reduced the oocyst viability of *Cryptosporidium parvum*. These findings suggest that silver nanoparticles were very effective and safe against parasitic infections of *Entamoeba histolytica* and *Cryptosporidium parvum* [137].

#### 5.1.4 Antibacterial activity

**Figure 4:**
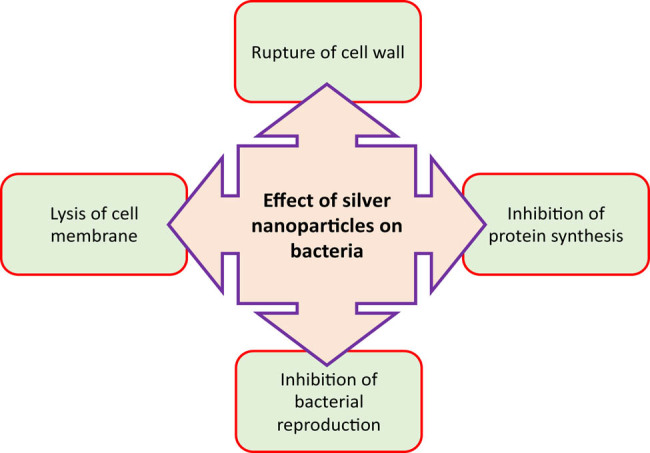
Diagram of antibacterial activity mechanisms of silver nanoparticles.

Silver nanoparticles play an important role as antibacterial agents. Silver nanoformulations have also been found to possess a good capability for inhibiting the growth of microorganisms such as bacteria (Figure 4) [138]. Silver nanoparticle-based devices are commonly used in dental and cardiovascular implants because they do not cause infections. It has been reported that silver nanoparticles have a powerful antibacterial activity against both Gram-negative and Gram-positive bacteria [139,140]. Some studies have reported that Gram-negative bacteria are more sensitive than Gram-positive bacteria to silver nanoparticles [140,141], whereas contradictory results were observed by other researchers [142]. They suggested that the differential sensitivity of both bacterial species could be attributed to the difference in their structural characteristics as well as the shape and size of silver nanoparticles.

Furthermore, it was reported that the antibacterial activities of various types of antibiotics were increased in the presence of silver nanoparticles [37]. The antimicrobial activities of silver nanoparticles against different pathogenic organisms were investigated by Nanda and Saravanan. The maximum antimicrobial activity was observed against methicillin-resistant *Staphylococcus aureus* [143]. Antibacterial and antibiofilm activities of silver nanoparticles alone and in combination with conventional antibiotics against various human pathogenic bacteria were examined. The findings of the study confirmed that in combination with antibiotics, there were noteworthy antimicrobial and antibiofilm effects which were seen at the lowest concentration of silver nanoparticles that were biosynthesised by using a plant extract of *Allophylus cobbe* [144]. Morones et al. indicated that size was an important factor affecting the bactericidal properties of silver nanoparticles [145].

Qasim and colleagues examined the antimicrobial activities of silver nanoparticles encapsulated in poly-*N*-isopropylacrylamide-based polymeric nanoparticles. The study revealed that the bacteriostatic activities of polymeric nanoparticles were determined by the size of nanoparticle as well as the amount of AgNO_3_ [146]. A pioneering study has discussed the antibacterial activity mechanism of silver nanoparticles [147].

#### 5.1.5 Antifouling action

It is known that biofouling is one of the major challenges faced by the water industry and public health. Silver nanoparticles of *Rhizopus oryzae* fungal species have been tested on contaminated water. Silver nanoparticles derived using *Lactobacillus fermentum* cells were found to control biofilm formation and were confirmed to have antifouling properties [91]. Moreover, silver nanoparticles are also applied to several types of environmental concerns such as air disinfection, water disinfection and surface disinfection [148]. A recent study demonstrated that an efficient management of biofouling can be achieved by a direct deposition of silver nanoparticle coatings on environmentally friendly surfaces [149].

#### 5.1.6 Antibiofilm activity

Worldwide, the food industry and community are subject to microbial biofilm challenges. Johani and colleagues conducted a study to evaluate decontaminated endoscope channels for residual bacterial contamination and biofilm presence. They demonstrated that 47% of channels were culture positive, with α-haemolytic streptococci from gastroscopes and coliforms from colonoscopes the most frequently isolated species. However, all 39 channels examined contained biofilm. Besides, it was observed that environmental bacteria were the chief components of this biofilm but potent pathogens were also present through all samples [150]. Due to the rapid emergence of antimicrobial resistance and the limited effect of antibiotics on bacterial biofilm, alternative strategies such as green silver nanotechnology are gaining attention due to the unique size, shape and structure of nanoparticles produced by this method.

Recently, people have started using silver nanoparticles for inhibiting biofilm formation, but the exact mechanism of the inhibitory action of silver nanoparticles is not clearly understood. Chen et al. categorised antibiofilm strategies into two groups: (i) treatments that inhibit the biofilm formation specifically and (ii) prevention and use of modified biomaterials in biomedical devices to make them resistant to biofilm formation [151]. Previous reports supported the new approaches for modification of the surface of biomedical devices to prevent microbial attachment, adhesion and growth [152]. In a study of the antibiofilm activity of silver nanoparticles against multidrug-resistant Gram-negative bacterial isolates, they effectively restricted biofilm formation [153]. Based on their findings, Martinez-Gutierrez et al. concluded that the formation of biofilms was prevented by silver nanoparticles and bacteria were killed in recognised biofilms [154].

Palanisamy et al. conducted a study to check the effect of silver nanoparticles on the formation of biofilm. They demonstrated that the formation of biofilms in resistant strains was inhibited by silver nanoparticles [155]. Another recent study was made to evaluate the contacts of silver nanoparticles with *Pseudomonas putida* biofilms. It was shown that treatment with silver nanoparticles suppressed the biofilms [156]. Kalishwaralal et al. investigated the antibiofilm activity of silver nanoparticles against biofilms formed by *Pseudomonas aeruginosa* and *Staphylococcus epidermidis*. The treatment of these organisms with silver nanoparticles showed inhibition of biofilm formation [157]. Mohanty and colleagues performed a study to check the antibacterial activity of silver nanoparticles against a panel of human pathogens. The data supported the previous studies that the biofilm formation was disturbed by silver nanoparticles. Furthermore, antibacterial activity was found to be improved, compared to a human cationic antimicrobial peptide [158].

### 5.2 Pharmacological uses of silver nanoparticles

#### 5.2.1 Wound healing

The biochemical events in wound repair are categorised into stages including inflammatory reaction, cell proliferation and synthesis of the elements that form the extracellular matrix and remodelling [159]. Silver nanoparticles either alone or in combination with antibacterial medicines are commonly used to promote wound healing without infection. Silver nanoparticle-based dressings have been applied to a fibroblast cell culture *in vitro* and to partial thickness burns in patients. A study has shown that silver nanoparticle-based dressings do not exert an influence on the proliferation of fibroblasts and keratinocytes that lead to a reestablishment of normal skin [160]. Besides, a combination of silver nanoparticles along with antibiotics such as tetracycline works more effectively than silver nanoparticles or tetracycline treatment alone against a bacterial load while wound macroscopic contraction was increased. Additionally, these findings suggest the use of a combination of silver nanoparticles and antibacterial medicines in the therapy of infected skin wounds [161].

Mats of gelatine fibres containing silver nanoparticles were prepared to form a wound-dressing pad by Rujitanaroj et al. [162]. A further study compared the efficacy of two antimicrobial agents including nanocrystalline silver and cadexomer iodine. In this study, community nursing clients with leg ulcers compromised by bacterial burden were chosen for a randomised-controlled trial. Their wounds were treated with either silver or iodine dressings. The results confirmed that treatment by using silver compounds was faster with a quick healing rate [163].

### 5.3 The use of silver nanoparticles in the food industry

Small quantities of silver nanoparticles are effective antimicrobials against bacteria and viruses but harmless to humans. This makes them useful for food sanitisation. Silver nanoparticles are used in widely available fresh food bags such as Sunriver Industrial Co. nanosilver food bags [114,118].

## 6 Other therapeutic uses

### 6.1 Antitumour activity

Cancers are multifactorial diseases, including alteration in cell signalling pathways. Natural products or active compounds of medicinal plants have a proven role in cancer prevention through killing of cancer cells. In this regard, silver nanoparticles are found to have an important role in cancer cell inhibition and thereby, inhibition of development and progression of the disease. It has been confirmed that silver and gold nanoparticles have a vital role in the inhibition of the growth of cancer cells. Studies based on lymphoma cell lines were performed to investigate the potential of silver nanoparticles as an antitumour agent *in vitro* and *in vivo*. The study confirmed the dose-dependent cytotoxicity of silver nanoparticles against lymphoma cells *in vitro* and also indicated a role in the induction of apoptosis. Additionally, it was reported that nanoparticles significantly increased the survival time in the tumour mouse model and also had a role in the decrease of the volume of ascitic fluid in tumour-bearing mice [164].

The effect of silver nanoparticles on gene expression in the human lung epithelial cell line was analysed. The study revealed that exposure to silver nanoparticles influenced the cell cycle and directed to an arrest in the G2/M phase [165]. It has recently been reported that silver nanoparticles induced autophagy in cancer cells through activating the PtdIns3K signalling pathway. Moreover, wortmannin, an inhibitor of autophagy, significantly enhanced the antitumour effect of silver nanoparticles in a melanoma cell model [166] and green synthesised silver nanoparticles showed a dose-dependent response based on the human lung cancer study [167].

The cytotoxic and oxidative effects of silver nanoparticles synthesised from *Panax ginseng* leaves were examined in human cancer cell lines. The study demonstrated that the nanoformulation had anticancer activity [168]. Khateef and colleagues examined the cytotoxicity of silver nanoparticles at various concentrations. It was noticed that the inhibition of cell growth was enhanced with increasing concentrations of the silver nanoparticles. Moreover, the increase in the concentration of silver nanoparticles led to decreased cell viability [169].

### 6.2 Drug-delivery systems

Drug delivery refers to methods of transporting natural or pharmaceutical compounds in order to attain a desired potential therapeutic effect. Various formulations based on nanoparticles have been reported to play an important role in drug targeting against various diseases. Polymers, such as microspheres and nanoparticles prepared from biodegradable compounds, have been reported to be used in drug targeting against disease processes such as inflammation and for cancer chemotherapy [170]. Hybrid molecular units holding silver nanoparticles are used to design drug-delivery systems to target inflammatory and infectious diseases [171,172]. Benyettou et al. synthesised a silver nanoparticle-based drug-delivery system to attain a simultaneous intracellular delivery of drugs such as doxorubicin and alendronate. This drug-delivery system has been shown to increase the anticancer therapeutic indices of both drugs [173]. Another study demonstrated that the hybridisation of Fe_3_O_4_ and silver nanoparticles can be used as high performance magnetic hyperthermia mediators [174].

### 6.3 Role in dentistry

Silver nanoparticles have been determined to have potential applications in dentistry through their ability to kill microbes or inhibit their growth. Moreover, the role of silver nanoparticles in areas including endodontics [155,156] and dental prostheses [175] has been noted. The potential use of silver oxide nanoparticles synthesised using *Ficus benghalensis* root extract has been reported and examined for its antibacterial activity against dental bacterial strains. The study demonstrated that a blend of the extract and Ag_2_O silver nanoparticles had powerful antibacterial activities [176]. Pérez-Díaz et al. reported that silver nanoparticles inhibited the growth of a planktonic *Streptococcus mutans* clinical isolate and killed *Streptococcus mutans* biofilms [177]. Santos et al. determined the bactericidal activity of silver nanoparticles against *Streptococcus mutans*. Thus, silver nanoparticles are suggested to have an effective role in the prevention of dental caries [178].

### 6.4 Orthopaedic implant/bone healing

In orthopaedic implants, silver nanoparticle-based devices are now preferred because of a lower risk of infection. Silver nanoparticle-coated stainless steel is used in order to reduce infections associated with orthopaedic implants. Structural characterisation of a unique hydroxyapatite (HAp) combined with silver nanoparticles has been examined for its application in orthopaedic implants and it was confirmed that it is well suited to orthopaedic implantation [179]. Another study has reported that silver nanoparticle-doped HAp scaffolds, showing a unique antibacterial activity, are able to prevent bacterial infections linked with bone implants [180]. In an experimental study, Ciobanu and colleagues obtained a novel HAp-based material with high biocompatibility. They demonstrated that viability was improved, and the activation of murine macrophages was potentiated by nanocrystalline silver-doped HAp [181].

### 6.5 Cardiovascular implants

Silver nanoparticle-based devices have proven applications in cardiovascular implants because of their antibacterial and anticoagulant activity. A recent study was performed to examine perfusion pressure and left ventricle pressure as physiological characteristics of cardiovascular function in response to silver nanoparticles. This study determined that hypertension strengthened the cardiotoxicity of silver nanoparticles [182]. Multilayer films containing nanosilver particles have been reported to function as antibacterial and anticoagulant agents. These multilayer films may have a good potential for surface modification of medical devices, particularly for cardiovascular implants [10].

## 7 Toxicity of silver nanoparticles

The use of silver nanoparticles is rapidly increasing worldwide in many sectors, including health. However, it is essential to minimise the risk of the adverse effect of silver nanoparticles on both human patients and the environment. In this regard, several studies based on animal models have been conducted to evaluate the toxicity of silver nanoparticles and their effect on physiology and tissue architecture. Ag^+^ leads to a non-classical permeability increase in the mitochondrial inner membrane. Moreover, in rat liver mitochondria, there was an increased permeability that caused mitochondrial swelling, abnormal metabolism and ultimately cellular apoptosis [183]. A further study found significant depletion of glutathione, decreased mitochondrial membrane potential and enhanced reactive oxygen species levels. These results suggest that in liver cells the cytotoxicity of Ag particles in the size range 15–100 nm is probably facilitated via oxidative stress [184].

A study was carried out to examine the suitability of a mouse spermatogonial stem cell line as a model to assess nanotoxicity and established a concentration-dependent toxicity for all types of particles tested, and silver nanoparticles were the most toxic in this regard [185]. In 2010, Laban et al. showed that both dissolved and particulate forms of silver caused toxicity to fish embryos [186]. Sung et al. have indicated that a prolonged exposure to silver nanoparticles induced changes in lung function as well as decreases in tidal volume and other inflammatory responses [187]. In a study on female mice exposed to various sizes of silver nanoparticles (10, 60 and 100 nm), the smaller silver nanoparticles (10 nm) showed the highest level of histopathological changes of congestion, single cell necrosis and focal necrosis in the liver and congestion in the spleen, suggesting that the smaller-sized particles showed higher acute toxicity in mice [188].

## 8 Conclusion

Silver nanoparticles play a significant role in health management due to their wide range of applications as antimicrobial agents, antitumour agents and in food packing, in agriculture and in the healthcare sector. Furthermore, it is well known that most of the empirical use of antibiotics exhibits resistance leading to ineffectiveness. Hence, biofilm forming bacteria present a serious problem. To overcome this problem of antibiotic resistance, there is increased worldwide attention on alternative treatment strategies. These alternative strategies include potential use of silver nanoparticles and surface coating or impregnation of nanomaterials as antibiofilm agents. In addition, silver nanoparticles are the most studied and utilised nanoparticles in the management of various diseases including cancer, wound healing, dental implants and other therapeutics such as modulating biological activities. With enhanced understanding and improved technology, the application of these novel particles in medicine will establish a standard platform for the prevention and treatment of multidrug resistance and biofilm pathogens.
